# Novel Method for Analysis of Allele Specific Expression in Triploid *Oryzias latipes* Reveals Consistent Pattern of Allele Exclusion

**DOI:** 10.1371/journal.pone.0100250

**Published:** 2014-06-19

**Authors:** Tzintzuni I. Garcia, Isa Matos, Yingjia Shen, Vagmita Pabuwal, Maria Manuela Coelho, Yuko Wakamatsu, Manfred Schartl, Ronald B. Walter

**Affiliations:** 1 Department of Chemistry and Biochemistry, Molecular Biosciences Research Group, Texas State University, San Marcos, Texas, United States of America; 2 Physiological Chemistry, Biozentrum, University of Würzburg, Würzburg, Germany; 3 Centro de Biologia Ambiental, Faculdade de Ciências da Universidade de Lisboa, Universidade de Lisboa, Lisboa, Portugal; 4 Comprehensive Cancer Center, University Clinic Würzburg, Würzburg, Germany; National University of Singapore, Singapore

## Abstract

Assessing allele-specific gene expression (ASE) on a large scale continues to be a technically challenging problem. Certain biological phenomena, such as X chromosome inactivation and parental imprinting, affect ASE most drastically by completely shutting down the expression of a whole set of alleles. Other more subtle effects on ASE are likely to be much more complex and dependent on the genetic environment and are perhaps more important to understand since they may be responsible for a significant amount of biological diversity. Tools to assess ASE in a diploid biological system are becoming more reliable. Non-diploid systems are, however, not uncommon. In humans full or partial polyploid states are regularly found in both healthy (meiotic cells, polynucleated cell types) and diseased tissues (trisomies, non-disjunction events, cancerous tissues). In this work we have studied ASE in the medaka fish model system. We have developed a method for determining ASE in polyploid organisms from RNAseq data and we have implemented this method in a software tool set. As a biological model system we have used nuclear transplantation to experimentally produce artificial triploid medaka composed of three different haplomes. We measured ASE in RNA isolated from the livers of two adult, triploid medaka fish that showed a high degree of similarity. The majority of genes examined (82%) shared expression more or less evenly among the three alleles in both triploids. The rest of the genes (18%) displayed a wide range of ASE levels. Interestingly the majority of genes (78%) displayed generally consistent ASE levels in both triploid individuals. A large contingent of these genes had the same allele entirely suppressed in both triploids. When viewed in a chromosomal context, it is revealed that these genes are from large sections of 4 chromosomes and may be indicative of some broad scale suppression of gene expression.

## Introduction

Allele specific expression (ASE) is an important component of gene regulation that is not well studied, but is thought to account for a major part of the phenotypic variation within and among species [1], [2]. Among plants in general, and particularly in many food crops, polyploidy also plays a major role in enhancing phenotypic variation and is often associated with increased vigor and the gain of desirable traits [3]. In plants made polyploid through hybridization, homoeologous genes (ancestrally homologous genes incorporated in an allopolyploid organism) can have uneven allele specific expression levels or overall gene expression levels that differ greatly from the parents [4]. These homoeologous genes bring together their accompanying regulatory elements which interact with the rest of the regulatory machinery upon hybridization to unevenly affect allele expression and may lead to extensively altered phenotypes [5]. In order to understand the impact of allopolyploidization on a molecular genetic level, it will be necessary to study ASE on a genome-wide scale.

In addition to better understanding of plants important to our food supply, understanding ASE in polyploid states is important to human health. In many cases cancerous cells contain multiple extra chromosomes leading to partial or full polyploidy [6]. Nondisjunction events also result in partially duplicated chromosomes and are mostly incompatible with life in humans, but in other cases lead to large phenotypic disruptions [7]. All of these situations are related to the more basic question of how ASE is affected by the elevation of a diploid genome to a polyploid state.

In such a situation it may be that alleles of each gene are expressed at the same levels in the polyploid environment as in the diploid such that the total gene expression is greater than that of a parent. Alternately there could be some form of dosage compensation such as silencing of individual alleles or of an entire haplome (genetically distinct set of chromosomes). There may be a bias for one haplome, or it could be random. In order to sufficiently answer these questions it is necessary to study the whole genome where all alleles can be distinguished, but this has proven to be problematic. It is a problem common to many polyploid biological systems and has been faced before largely in plant studies but also some animals [5]–[9].

Several studies have addressed the basic issue of ASE in polyploid systems in plants, and a recent review provides an excellent summary of these findings [5]. In general a wide array of expression patterns have been observed in hybrid systems [5], [8], [9]. In some cases expression from each allele may be additive. For other genes, however, the expression is dominated by one allele. These effects may be tissue-specific, responsive to environmental cues, or they may be biased toward one parent. In general these observations have been made on small gene sets. High throughput studies include some use of microarrays [10], [11] and a recent analysis of allopolyploid (AD) cotton using RNAseq [4].

Examples of polyploid animals are rare especially among vertebrates, but several examples exist among amphibians and teleost fish. An especially notable example is that of the *Squalius alburnoides* complex. This is a naturally occurring inter-generic hybrid population descended from *Squalius pyrenaicus* females and males of an extinct species in which individuals may contain between 2–4 genome copies [12], [13]. A mechanism of dosage compensation is employed by this fish is the silencing of specific alleles, but not of an entire haplome [12].

The ability to measure ASE in these situations is vital to better understand global mechanisms of allele dosage compensation and to disentangle gene interaction networks. Thus, we have generated a model in which three haploid genomes with sufficient genetic diversity to allow determination of allele specific gene expression were combined to experimentally produce triploids. We used the small laboratory fish medaka (*Oryzias latipes*) to produce two triploid individuals through nuclear transfer of a diploid F_1_ nucleus to a recipient ovum, thus incorporating haplomes from three disparate medaka strains. We then developed a computational methodology to derive allele-specific expression values from RNAseq data obtained from isolated liver RNA from the triploid and parental diploid fish. In the triploid medaka, we find that alleles are expressed at similar levels in most cases, but allele suppression is not uncommon and occurs consistently in the two triploid fish. In some cases, the suppressed alleles are completely silenced and in these cases the silenced allele is almost never derived from the maternal genome component stemming from the mother of the F_1_.

## Results

**Figure 1:**
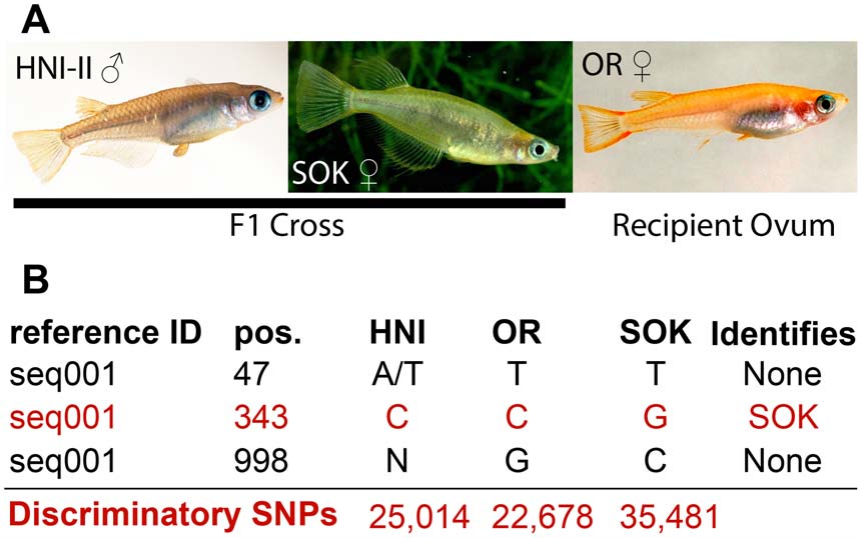
Parent strains and variant calling. (A) Parent strains and gender of donor genomes (images provided by MS). HNI-II males were mated with SOK females to produce F_1_ embryos. At the blastula stage, cells were separated and diploid nuclei from them were injected into OR ova where they fused with the haploid nucleus of the oocyte. (B) Examples of variants called by VarScan. Only variant positions in which at least one strain was completely and unambiguously different from the others could be used (variant at position 343). Variant positions were unsuitable where one strain was only partially different (i.e. from heterozygosity) or where a strain had insufficient coverage to confidently call a set of observed nucleotides (variants at positions 47 and 998). The total number of dSNPs identified for each strain is indicated in the bottom line of red text.

In order to produce artificial triploid fish, diploid hybrid F_1_ embryos were first produced through the natural mating of two genetically different strains (the Houiken Niigata-II, or HNI-II, strain originates from a wild population in northern Japan and the Sokcho, or SOK, strain originates from a wild population in east Korea). Then diploid hybrid nuclei from the developing embryonic blastomeres were transplanted into unfertilized eggs of a third medaka strain (the orange-red or OR strain is derived from a commercially available variety originated from a southern Japanese wild population). The resulting triploid embryos have three genetically distinct sets of chromosomes (haplomes). Through this technique two triploid fish were produced for this study (from now on referred to as trpA and trpB) that incorporated genetic material from three divergent strains of medaka (Figure 1A). Both individuals were phenotypically female, and developed as apparently healthy adults, but were infertile.

### Determination of dSNPs

To distinguish alleles and determine the contribution of each allele to overall gene expression we focused on using SNPs identified in the three parental strains that had been used to produce the triploids. Similar approaches for diploid organisms have been successful [14]–[16]. In diploid organisms SNPs can be used to discriminate expression levels of two alleles, but in the triploid case it is uncommon for a single SNP position to discriminate all three alleles. Instead we identified SNPs that were found to have only one possible nucleotide in one strain that did not overlap with the observed nucleotide possibilities in the other two strains at the same position in the same transcript.

Short read data for the three parental strains were aligned using the STAR aligner [17] (https://code.google.com/p/rna-star/). The STAR aligner was utilized since it is fast and can accurately handle several mismatches, indels and/or splice junctions. Since the reference sequences were transcripts, the ability to align over splice junctions was disabled, but alignment over short indels was still allowed. Short read samples had between of 54 to 61% of the reads alignedalign (Table 1). The reference sequences in ENSMBL arewere largely based on high throughput sequencing of the Hd-rR strain of medaka which, like the OR strain, also originates from the southern Japanese population. This was further supplemented with data from the HNI strain that is similar to the HNI-II strain and also originates from northern Japan. Therefore it is nottherefore surprisingsurprise these two strains had the most reads aligned and fewest dSNPs and indels detected. The SOK strain, originating from south Korea has the fewest reads aligned and most dSNPs and indels. Even so, using the STAR short read aligner that can work around mismatches and indels, the number of SOK reads aligned is not drastically different from the number of OR and HNI-II reads aligned (Table 1). We detected 9,913 putative indels throughout the full set of transcripts. The indels in general did not seem to be very large (typically between 2–7 bp and up to 21 bp).

**Table 1:** Short read data and variants detected per sample.

Sample	Gender	Pre-filter fragments	Post-filter fragments	Aligned Fragments	Percent Aligned	dSNPs	indels
HNI-II	M	7.50E+07	7.20E+07	4.07E+07	57%	25,014	5,772
OR	M	6.30E+07	6.00E+07	3.68E+07	61%	22,678	3,337
SOK	M	6.10E+07	5.90E+07	3.19E+07	54%	35,481	6,085
TrpA	F	2.10E+08	2.00E+08	1.11E+08	56%	n/a	n/a
TrpB	F	2.00E+08	1.90E+08	1.13E+08	60%	n/a	n/a
synthetic	n/a	n/a	†1.11E+08	1.11E+08	100%	n/a	n/a

Short read data for the five samples was run in three lanes on the Illumina HiSeq 2000 platform resulting in roughly 200 million read pairs per lanelane (data available from sequence read archive associated with BioProject accession: PRJNA246137). During our filtration process it is possible some reads lose their mate while in cases where the reads overlap significantly, they are merged into one read. Therefore it is more useful to use the term fragments (the sum of pairs and single reads) as opposed to reads or pairs. In the range of 54–61% of fragments aligned successfully to our transcriptome reference sequences. The synthetic data set having been generated exclusively from the reference sequences aligned completely.

† The synthetic reads were not filtered, this number is the total number of synthetic reads generated.

Using the set of cDNA records from the ENSEMBL 65 medaka genome annotation as our reference, we initially detected 250,982 single nucleotide variant positions where at least one of the three parent strains differed from the reference sequence. These positions however included many that did not provide useful information for the following reasons: one or more strains are heterozygous such that none can be completely distinguished from the other two (Figure 1B, position 47), the coverage in one strain is too low to make a confident call of observed sequence (annotated by VarScan as an N; Figure 1B position 998), or all three strains agree with one another but disagree with the reference. Excluding these cases left 109,581 informational sites which can distinguish the expression of one allele from the other two. For the sake of brevity, and convenience, we call these sites discriminatory SNPs (dSNPs) throughout this report. In order to measure ASE values for all three alleles in a gene, the dSNP number was further reduced to a final total of 83,173 dSNPs that occurred in transcripts containing at least one dSNP representative of each parental strain (Figure 1B discriminatory SNPs). The reference sequences we used were primarily based on data from the OR strain, and thus this strain had the fewest dSNPs detected.

Another departure from normal diploid determination of ASE is that each parental strain had a different set of dSNPs that distinguish it, and therefore we cannot simply count the reads attributable to each parental haplome. This is largely due to the wide variability in the number of dSNPs for each parental strain for some transcripts. For example, in a given reference transcript one haplome may be represented by only 1 or 2 dSNPs while another may have 20; this situation would bias read counts toward the haplome having 20 dSNPs. A second reason for not performing standard read counting is the large variability in coverage depth possible over the length of a transcript. Each dSNP can only sample the expression signal from one haplome at one position along the transcript. Thus, it may be misleading should the position of the dSNP coincide with a very low or very high depth of coverage for a given transcript.

Our strategy was to use the depth of coverage information at dSNP positions to identify the fraction of that coverage depth that was attributable to the haplome for which each dSNP was specific. Then the coverage fractions attributable to each haplome were integrated to give a single ASE value for that haplome. In order to derive ASE values from these single positions, fractional expression values are combined from dSNPs in the same gene. We were therefore constrained to the 4,282 transcripts (of 24,662 annotated in ENSEMBL version 65) that had at least one dSNP representative of each of the three haplomes. The final set of 83,173 dSNPs are present in these 4,282 transcripts resulting in an average of 19.4 dSNPs per transcript.

### Determination of ASE from Normalized Coverage Depth

**Figure 2:**
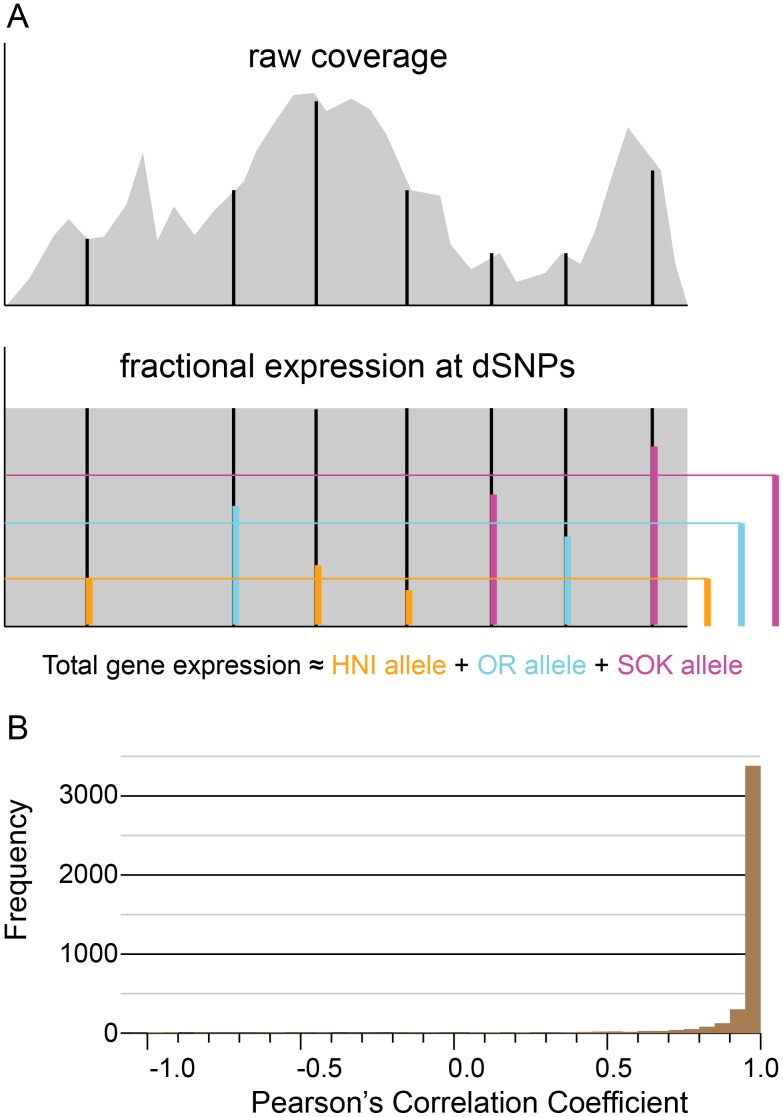
Illustration of ASE method and analysis of artificial data. (A) Cartoon of the raw coverage observed when RNAseq reads are mapped to an example mRNA transcript and the effect of normalizing coverage across the transcript. In both raw and normalized coverage plots, black vertical lines indicate the positions of dSNPs. In the cartoon of normalized coverage, the colored vertical bars at dSNP positions indicate the contribution of the discernible allele to the overall expression, and the colored bars to the right indicate the average of expression values measured at dSNP sites for each allele. (B) Correlation of known ASE values to calculated ASE values for the synthetic data test. The calculated allele-specific expression values are compared with the original known values for each transcript by calculating a Pearson’s correlation coefficient. This will measure how well the trend in the calculated ASE values matches the trend in the original ASE values. Over 75% of the transcripts (2,628) had a correlation coefficient greater than 0.8.

Short reads aligned to a reference transcriptome generally result in uneven depth of coverage (Figure 2A raw coverage). Many factors may contribute to this phenomenon and it is commonly observed in all RNAseq data. For example the expression of alternative splice forms, where two expressed splice forms may provide the common exons and thus more reads in an additive fashion, while differently incorporated exons would only be expressed at the level of each splice form. Additionally, annotated genes often include only some of the splice forms or possible exons that actually make up a locus thus making an accurate measure of gene expression more difficult. Another factor that may affect coverage variability in some transcripts stems from the inability of short read aligners to distinguish between equally good alignment locations. Some subsequences of the reference can be very common and thus give the short read aligner a difficult choice. In these situations it is common to simply not report alignments to these regions, to randomly select one, or to report alignments to all regions. Thus, choices made in these areas can result in more or less reads aligned to them. To circumvent this problem in our analyses we chose to assess the fractional expression at dSNP sites in each transcript (Figure 2A fractional expression at dSNPs). Herein the fractional expression values were multiplied by the geometric mean of coverage depth of the transcript in which they occur in order to obtain an allele specific expression value for each dSNP position. We chose the geometric mean of coverage depth as a measure of gene expression in order to mitigate the effects of transcript length and lessen the bias in apparent expression value due to spikes in coverage. The dSNP expression values specific to each strain are then averaged in each transcript to arrive at an allele specific expression (ASE) value for each allele in the transcript (Figure 2A fractional expression at dSNPs). The ASE values calculated may be found in Table S3.

### Synthetic Data Test

To evaluate the ability of this technique to accurately establish allele specific gene expression values, we devised a set of synthetic data in which these values were known. We generated an artificial set of short read data from the full set of medaka transcripts. To generate these synthetic reads, we produced a strain-specific set of transcript sequences wherein the strain-specific SNP nucleotides were substituted into the reference sequence. The total number of reads generated for each transcript was the same as that found by mapping short reads from trpA to the reference sequences, and we kept the allele balances consistent with those measured in trpA. For 91% of the transcripts in this data set (3,891 out of 4,282), we obtained a correlation of greater than 0.8 when comparing measured to actual allele expression values, with the bulk of these (3,684, 86%) having a correlation coefficient of 0.90 or greater (Figure 2B).

We ruled out several factors that were speculated to adversely affect the analysis in these 9% of cases including overall expression, numbers of dSNPs per transcript, and frequently found sub-sequences. The details of our efforts are given in Text S1. The randomness of generated reads likely had a role in the poor correlation of some transcripts in cases where the three alleles had known values very close to one another. Presume for example the known values were 255, 260, and 265 for the three alleles. These quantities of reads would have been generated from random locations along the length of the strain-specific references to represent each allele. Some noise therefore is introduced and the reconstructed ASE values may have been 11, 12, and 11. A reasonably close result which nevertheless results in a correlation coefficient of 0.

### Validation of SNP Calls and ASGE Trends in the Triploid Fish

**Figure 3:**
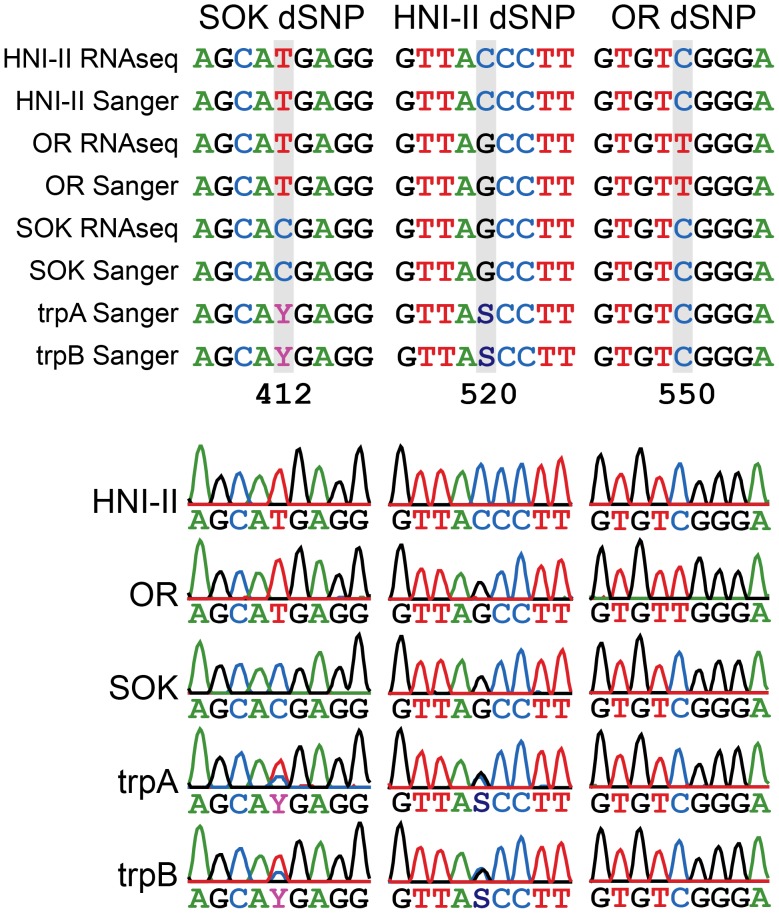
An example of parental dSNP and tri-hybrid ASGE validation. The represented transcript is ENSORLT00000013099 (ensembl Transcript ID). One dSNP per parental genome is represented. (A) RNA-seq and Sanger obtained sequence alignments between the parental strains (SOK, HNI-II and OR) and the Sanger obtained sequences for both trpA and trpB triploids. (B) Aligned chromatograms of the three parental strains and trpA and trpB. In both triploids the allele expression pattern determined by Sanger is consistent with the one obtained by RNA-Seq for this transcript. In this example SOK and HNI-II dSNPs are observed in the triploid, but the OR dSNP is not observed.

We selected seven genes with extreme allelic expression patterns for validation (Table S1 and Figure S1). We performed Sanger sequencing of the PCR products for each target gene in the triploid fish and for the three parental strains. All but two of the 36 SNP calls at dSNP sites were validated in all seven sequenced transcripts (Table S2). The trends in usage of specific alleles in triploid fish was also assessed using a previously published method [12], [18]. The trends in allele-specific expression observed by Sanger sequencing were also consistent with our RNAseq-based ASE method (Figure 3 and Table S2).

### The Geometric Mean of Coverage Depth Compares Well to Read Counting

**Figure 4:**
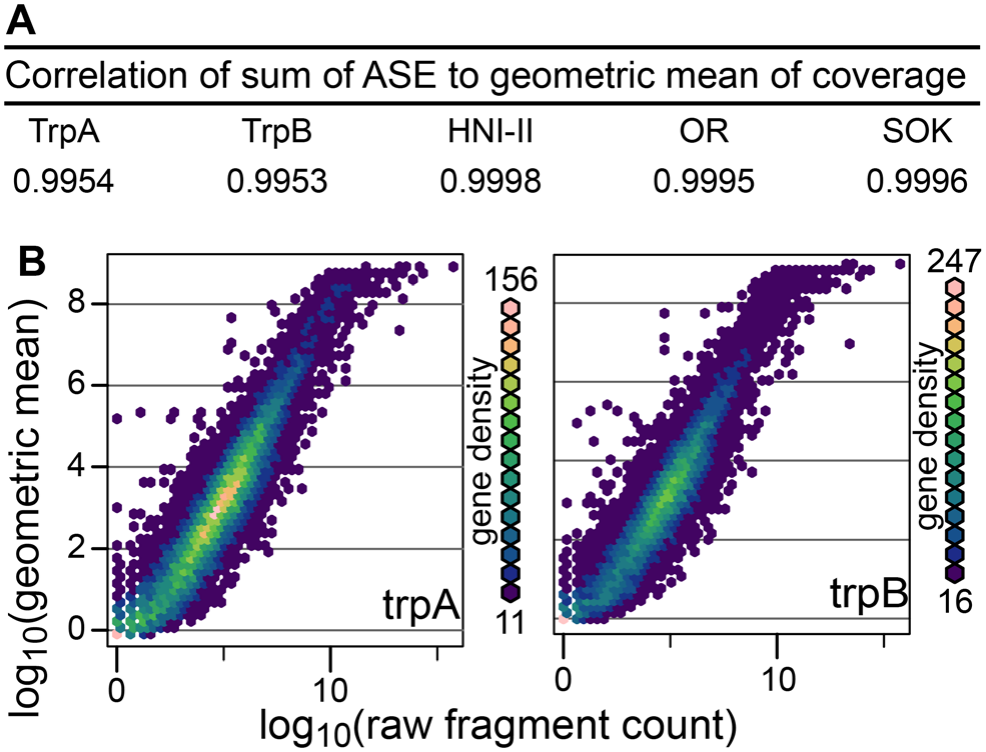
Comparison of calculated ASE values to whole gene expression. (A) Correlation of sum of ASE values per transcript (calculated only from dSNP sites) to geometric mean of each gene (calculated from coverage over entire transcript). (B) Plot of geometric mean of read coverage against raw fragment count showing a strong correlation between the two.

We now discuss exclusively RNAseq reads from the biological samples aligned to the set of 4,282 ASE-compatible medaka transcripts. We are using the geometric mean of coverage in each transcript as a measure of the total expression of that transcript. Each dSNP gives us the fractional expression of one allele measured from the coverage depth at that position. These fractional expression values are averaged over all dSNPs specific to each strain in order to arrive at a final ASE value for the three strains. We observe a strong correlation between the sum of calculated ASE values and the geometric mean of the coverage in each transcript (Figure 4A). This indicates the method employed accurately divides the overall expression between the three alleles.

In order to examine whether the geometric mean of coverage depth was a good measure of gene expression, we compared it to the commonly used method of counting fragments mapped to each transcript. The Spearman’s correlation was calculated comparing the whole gene expression values calculated by the two methods. A very high correlation is observed between the two triploids (trpA r = 0.941, trpB r = 0.938 see Figure 4B). We chose to use the Spearman’s correlation because, as shown in Figure 4B, the rate of increase in the normalized expression values is reduced as higher fragment counts are reached. This is likely due to the normalized expression value being insensitive to the length of the original transcripts whereas the read counting method produces higher values for longer transcripts.

### A Major Set of Allele-imbalanced Genes have Silenced One Allele

**Figure 5:**
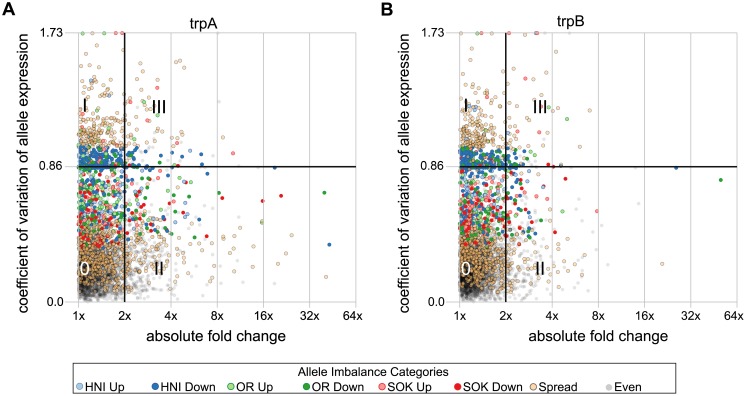
Overall gene expression compared to the dispersion in allele specific expression. A comparison of the change in gene expression with the dispersion of allele specific expression in trpA and trpB are plotted in panels (A) and (B) respectively. The horizontal axes indicate differential expression of the triploids with respect to the parent fish lines. The average of whole gene expression in trpA and trpB is compared to the average of the 3 parent species. The vertical axis of (A) indicates the coefficient of variation (c_v_) of ASE values for each transcript in trpA, while (B) indicates the same quantity for trpB. c_v_ values near 0 indicate that the three alleles are expressed at near equal levels, and increasing c_v_ values indicate a greater dispersion of allele-specific expression. A line at a c_v_ value of 0.86 is drawn because this value correlates with the situation where one allele is shut down entirely and the other two are expressed at similar levels to one another. Similarly the line at a c_v_ value of 1.73 correlates with transcripts in which two alleles have been shut off. Additionally, a grid is drawn to separate the plot into four quadrants. Quadrant ‘0′ has transcripts in which ASE values are least dispersed and gene expression is similar to the average of parental strains. Quadrant I contains transcripts which are expressed at levels similar to those in the parents but in which ASE levels are more highly dispersed. Quadrant II contains transcripts in which expression levels are dissimilar to the parents, but with low ASE dispersion. Quadrant III contains transcripts in which expression levels were dissimilar to the parents and ASE dispersion was increased. Points are colored to reflect categories defined by the balance of allele expression in each transcript (allele imbalance categories).

Since we only have two individuals in our analysis we selected two arbitrary boundaries in order to help interpret the data. We are interested in dividing genes into groups that may be differentially expressed in the triploid environment from those that seem to be expressed at similar levels to the average of parental expression. To this end we select a threshold of 2-fold change either up or down in gene expression for each triploid with respect to the average parental gene expression. The coefficient of variation (c_v_) is used here as a normalized measure of dispersion of the allele specific expression values. A low c_v_ indicates the three alleles for a given gene have relatively equal expression to one another. As the c_v_ rises the expression of the three alleles becomes more divergent. The highest possible c_v_ is 1.73 which corresponds to the condition where one allele shows some expression while the other two alleles are totally shut down. The data indicate a significant clustering of genes have a c_v_ of ∼0.86. This corresponds to the situation in which one allele is not expressed (zero or near-zero expression levels) while the other two alleles make up the bulk of expression. We use this as a second arbitrary threshold to divide our data. These boundaries are shown in four quadrants of plots in Figure 5 A and B. Quadrant 0 represents transcripts in which ASE values are relatively equal to one another and in which the overall gene expression is similar to that of the parental strains (3,166 transcripts in trpA and 3,415 transcripts in trpB with 2,918 shared between them). Quadrant I represents transcripts in which ASE values are more highly dispersed but in which the overall expression values are similar to the average of the parental strains (468 transcripts in trpA and 440 transcripts in trpB with 309 shared between them). Quadrant II represents transcripts in which overall expression differs from the parents but in which the ASE values are similar to one another (533 transcripts in trpA and 338 transcripts in trpB with 149 transcripts shared between them). Quadrant III holds transcripts with dispersed ASE values and in which overall expression has changed from parental strains (115 transcripts in trpA and 89 transcripts in trpB with 38 shared).

The distribution of coefficients of variance has a main peak (c_v_≈0.2) corresponding to transcripts with alleles that express at roughly equal levels in a triploid individuals (Figure S1). There is also a second peak (c_v_≈0.86) that corresponds to transcripts in which one allele does not appear to be expressed (Figure S1).

### ASE Imbalance Categories are Similar in both Triploid Fish

Allele specific expression values for each transcript were analyzed to identify allele expression imbalances that may indicate exceptionally high or low expression of one allele with respect to the other two. We first applied a goodness of fit test where the null hypothesis is that alleles express equally. This resulted in 1,593 transcripts in trpA and 1,447 transcripts in trpB for which the resultant p-value was less than 0.01. These sets of transcripts which deviate from equal allele expression were then broken down into groups indicative of exceptionally high or low expression of one allele.

**Figure 6:**
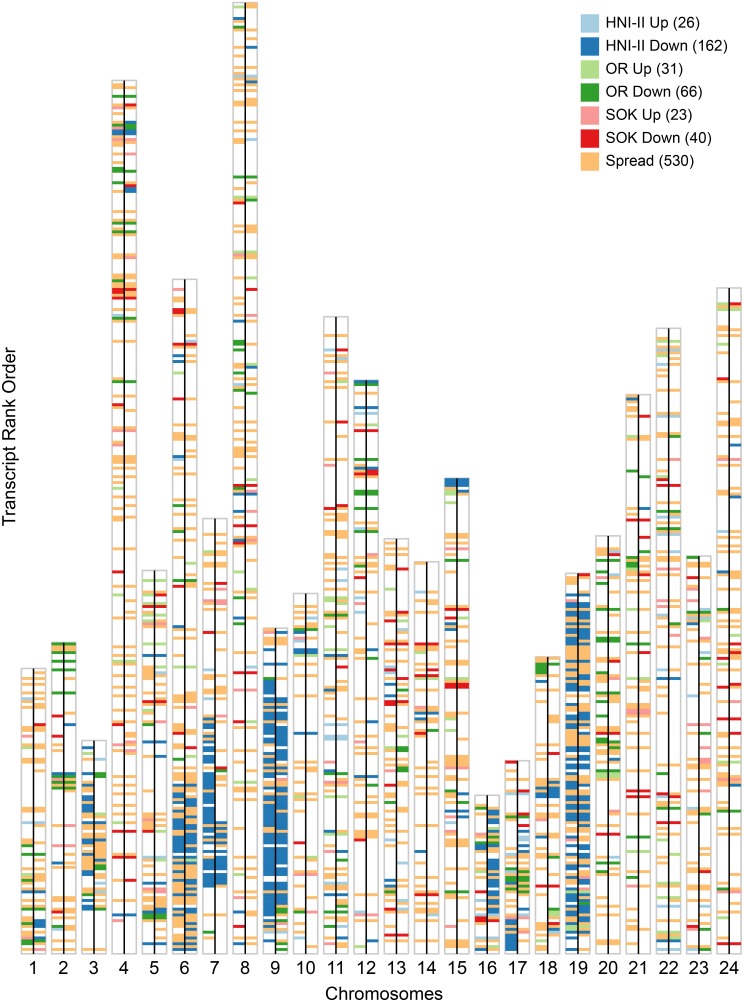
dSNP-complete transcripts which can be placed in chromosomes. Each chromosome is represented by large vertical white bar outlined in gray. Transcripts are represented by horizontal bars of uniform size and are placed in the rank order in which they occur in each chromosome. The bars are colored to indicate the allelic imbalance category to which the transcript belongs based on exceptional high or low expressing alleles. Blank spaces represent transcripts that could not be said to deviate from equal allele expression. Each chromosome is divided into a left and right half by a black line. The left half of each chromosome gives the plot for trpA, while the right half gives the plot for trpB. The tallest bar (that for chromosome 8) is comprised of 329 transcripts in the order they occur on the chromosome with ties being assigned sequential ranks. The total numbers of transcripts that fall into the same category in both trpA and trpB are indicated in parenthesis after each category name in the legend.

In order to identify high or low expressing alleles, we chose to use the median of allele expression in each transcript as a basis for comparison since it is more resistant to outliers than the median. This is especially true in cases where the number of observations is small and a single outlier will have a very strong effect on the mean. We selected an arbitrary boundary of 2-fold above or below the median as a threshold for selection as a high or low expressing allele. Six separate categories indicate one of each of the three alleles were expressed at 2-fold above, or below the median expression of the three alleles in a given gene (these categories are: HNI Up, HNI Down, OR Up, OR Down, SOK Up, and SOK Down). Another ‘spread’ category indicates genes have deviated from the expectation of equal expression, but not been incuded into another category, and the final ‘even’ category contains transcripts for which deviation from equal expression was not rejected (legend of Figures 5 and 6).

These groups help to identify trends in the distribution of c_v_. In both trpA and trpB the second peak (c_v_≈0.86) is primarily composed of transcripts in the HNI-Down category followed by those in the OR-Down category (Figure S2). Of the 4,282 transcripts analyzed, 358 were found to have one allele suppressed 2-fold below the median in both triploids, while 104 were found to have an allele expressed 2-fold higher than the median allele expression in both triploids. When genes in these categories are displayed in their chromosomal context, strong similarities appear between the two triploid fish (Figure 6). In fact 3,353 transcripts are in the same category in both triploids.

## Discussion

### Allele-specific Suppression is a Common Feature

Overall, most genes seem to be expressed at similar total levels in triploid fish as those in the parental strains. In the case of an imbalance the data suggest it is more common for one allele to be suppressed than it is for one allele to dominate expression of the gene (Figure 5 and Figure S2). A similar feature in which certain genes are effectively diploidized has been observed in triploid individuals of the naturally occurring intergenic hybrid *S. alburnoides* complex [12], [13], [18]. There does not seem to be a global preference for any given allele in transcripts based on either differential gene expression or allele specific gene expression. On the other hand, in both triploids, a majority of transcripts wherein one allele has been suppressed to expression levels at or near zero (Figure 5 and Figure S2 coefficient of variation near 0.87) are in only two categories: HNI down and OR down (Figure S2). Whatever mechanism is active in this regulation, it appears as though it has a bias for the donor female-derived haplome. In both triploids the male-derived haplome (HNI-II) is the most drastically suppressed and it is followed by the haplome of the recipient ovum (SOK). On the contrary, the donor female-derived haplome (OR) was nearly unaffected by drastic allele suppression. This could be reflective of some form of allele suppression that favors the donor maternal genome. This may be somehow associated with the different packing states of the chromatin since the diploid nucleus taken from the blastula cell had been in an active state whereas the chromatin of the recipient ovum may have still been in a more dormant and packed form. Thus it may be that the donor female haplome was able to impose some allele silencing regime on the male haplome and the recipient female haplome.

### ASE Imbalances are Similar between Triploid Fish

ASE analysis of two experimentally produced medaka triploids shows a consistent pattern of allele-specific gene regulation between the two triploid individuals. Of the 4,282 transcripts in our analysis, 3,353 are in the same ASE imbalance categories in both triploid fish (Figure 6). This implies common regulatory mechanism(s) may direct ASE in both fish. This is surprising since polyploidy is not commonly found in medaka populations, thus there is no expectation for the existence of selective pressures to develop a regulatory mechanism that would act consistently on ASE in the medaka genome in a polyploid state. In some cases in plants particular crosses have predictable patterns of ASE dominance/suppression [3] and in *S. alburnoides* the pattern of suppression is different between, yet consistent within, geographical populations [13]. In both of these cases, however, the similarities are due to preferential suppression of one whole haplome whereas we do not see evidence of this in the medaka triploids.

Some chromosomes have large regions in which one allele is predominantly affected in a similar manner. Specifically, chromosomes 6, 7, 9 and 19 each have large regions in which most of the allelic imbalances observed resulted from suppression of the HNI allele in both triploid fish (Figure 6). We explored the possibility the four chromosomes might have been related through duplication in the teleost-specific whole genome duplication event [21]. However, according to a recent analysis, these chromosomes are thought to have originated from separate ancestral chromosomes [22], so it is unlikely that they have a common set of ancestral regulatory regions. One possible explanation is that the triploid fish are genetically very similar and so the possible regulatory schemes are likely limited. The three parental strains are genetically very homogeneous due to the closed colony breeding in which genetic bottlenecks also occur. Additionally these artificial triploid fish could share a common parentage. Donor blastula cells and host ova were pooled during the nuclear transfer proceedure and it is possible the donor nuclei for both triploids were derived from sister blastula cells leading to a very similar set of genetic material from both HNI-II and SOK strains.

### Improvements Could Expand the Available Gene Set

In the current study we limited ourselves to producing ASE values for transcripts in which each allele was represented by at least one SNP. We were thus limited to a set of 4,282 transcripts out of 24,662 possible transcripts representing 4,181 of 19,687 genes that were annotated in the medaka genome (ENSEMBL version 65). One way it may be possible to expand the data set is to incorporate cases where one allele lacks dSNPs. In this case the ASE value of the remaining allele can be extrapolated from the calculated values of the two alleles with dSNPs. Doing this would expand the number of transcripts in our analysis by 1,728 or 40%.

One of the limitations on the number of transcripts amenable to ASE analysis was the variant calling step. We set very conservative thresholds when calling consensus sequences using VarScan, which likely precluded many lowly expressed transcripts from inclusion into our analysis. It is likely that many more variants could be reliably identified using an approach that specifically targets the genome and provides more even coverage, such as exome sequencing [19]. With a more robust and complete set of SNPs, it should be possible to significantly increase the number of genes in the analysis.

Another limitation of our method is that RNA-seq reads from three divergent strains were aligned to one common reference. This may cause a bias in relative expression levels since too many sequence differences occuring near one another may be a barrier to read alignment for those that do not originate from the same species or strain as the reference [20]. We ultimately used the STAR aligner which is permissive and can accurately align short reads over mismatches and indels and this aligner proved capable of greatly increasing the number of dSNPs able to used compared with others we employed (i.e., Bowtie).use. Additionally using the STAR aligner we found the balance of overall allele expression to be nearly equal (Figure S3) with only slight biases remaining against the most distant medaka strain. A more accurate yet more restrictive procedure is outlined in a recent report by Stevenson et. al. where only sites without such clusters of sequence variants are considered for ASE [20].

In summary, this complex data set has revealed several interesting biological features of the molecular-genetic activities of experimentally produced triploid medaka. The data made available by RNAseq based polyploid ASE analysis provide a highly detailed basis for the future analysis of genetic regulatory networks. This was enabled by the method we describe here for determining allele-specific expression in polyploid organisms on a large scale. Much of the software developed was created in such a way as to accommodate any ploidy number so as to be applicable to the more common diploid and/or the rare/exceptional higher-ploidy organisms with little modification. We expect this will expand our ability to understand the importance of ASE in other biologically and medically interesting systems.

## Methods

### Ethics Statement Regarding Animal Subjects

The research presented here complies with the applicable EU and national German legislation governing animal experimentation, especially the Tierschutzgesetz der Bundesrepublik Deutschland (German Federal Law of Animal Protection). The institution at which animal experiments were carried out is controlled by the Tierschutzbeauftragte (Animal Protection Officer) of the University of Wu?rzburg, Dr. Wolfgang Geise (Stabsstelle Arbeits-, Tier- und Umweltschutz, Marcusstraβe 9-11, D-97070 Würzburg), and therefore by the Veterinary Office of the District Government of Lower Franconia, Germany (Authorization number: 55.2-2531.01-49/08). Animal research conducted under this study has been approved by the institutional animal care and use committee of the University of Würzburg.

### Parental Strains

Three strains were used as parents to generate the allotriploid fish (Figure 1A). The OR (orange-red) strain of medaka, *Oryzias latipes* [23], is derived from a commercially available orange-red variety that primarily originated from a southern Japanese wild population. The SOK (Sokcho) strain of medaka [24], originated from a wild population in east Korea. The HNI-II (Houiken-Niigata-II) strain of medaka [25] (recently taxonomically described as separate species, *Oryzias sakaizumi*) is a strain originating from a wild population of the north of Japan. All three strains were maintained as closed colony stocks and propagated in the aquarium facilities of the Biocenter in the University of Würzburg under standard conditions [26].

### Donor Cells and Recipient Egg Preparation

F_1_ embryos were obtained from crossing males of HNI-II with females of SOK. Donor cells were obtained from these embryos. Eggs from OR females were used as recipients. Donors and recipients were prepared according to Niwa *et al*. [27]. Briefly, 20 to 30 donor F_1_ embryos at the early blastula stage were dechorionated with medaka hatching enzyme solution. Their blastoderms were dissociated into single cells. The cells were then collected by centrifugation and stored until use (up to 6 h) at 4°C in a buffer solution containing 0.25 M sucrose, 120 mM NaCl, 0.5 mM spermidine trihydrochloride (Sigma, St. Louis, MO), 0.15 mM spermine tetrahydrochloride (Sigma) and 15 mM HEPES (pH 7.3). Mature unfertilized eggs were collected from the ovary of female fish and kept in a balanced salt solution (BSS) for medaka [28] at 18°C until use (up to 5 hrs).

### Nuclear Transfer

Nuclear transfer was performed according to Niwa *et al*. [27] with small modifications. An oil pressure injector made by the technical department of University of Würzburg connected to a micromanipulator (MM 33, Märzhäuser, Wetzlar, Germany) was used along with a stereomicroscope (MZ16F; Leica, Weltzlar, Germany). Also, the entire procedure was performed at 7°C. Six days after the nuclear transplant, normally developing embryos were dechorionated with medaka hatching enzyme solution and kept at 26°C in BSS supplemented with 100 units/mL penicillin +100 µg/mL streptomycin [29] until hatching. Hatched larvae were reared normally to the adult stage.

### RNA Isolation

The liver of two triploid female medaka and the liver of one male of each parental strain (HNI-II, SOK and OR) ware collected in RNAlater (Qiagen) and used thereafter for RNA isolation. Total RNA was obtained with the RNeasy Mini Kit (Qiagen) and DNase treated on-column with the RNase-free DNase Set (Qiagen). Evaluation of integrity and quantification of the extracted RNA was performed with Nanodrop 1000 (Thermo Scientific) and 2100 Bioanalyser (Agilent Technologies) equipment. All five samples presented a RIN value of above 9 (Bioanalyser). RNA was divided in aliquots of at least 15 µg per sample and stored in RNAstable TM tubes (MoBiTec) at −80°C until further processing.

### RNA Sequencing

RNAseq library build and sequencing steps were performed at Expression Analysis (Durahm, NC). Purified, poly-A selected liver RNA from each of the two triploid individuals were sequenced in one lane each of an Illumina HiSeq instrument. RNA from the three parental lines was multiplexed into a third lane, and all three lanes were sequenced as 100 bp paired ends. The resultant short reads were filtered for quality using a custom filtration pipeline. In general, steps include removing adapter sequence, trimming away low quality regions, and merging overlapping reads. Less than 5% of reads were lost during filtration and the parental strain short read libraries each consisted of approximately 64 million reads, while each of the triploid fish libraries consisted of around 200 million reads (Table 1). Short read libraries have been deposited in the sequence read archive (SRA) under BioProject accession: PRJNA246137 (https://www.ncbi.nlm.nih.gov/bioproject/).

### SNP Calling

Reads from each of the three parental strains were separately aligned to the reference transcript sequences (Medaka cDNA ‘all’ not ‘*ab initio*’ records from ENSEMBL v 65) using STAR version 2.3.0e (linux 64-bit pre-compiled binary) [17] to produce output files in the SAM format which were converted to sorted BAM files using samtools (Protocol S1). Each sample was aligned separately to produce three output files in total. These files were then converted to BAM format, sorted, and finally converted to mpileup format using samtools version 0.1.18 [30]. These mpileup files were then inputs for the VarScan (version 2.3.6 varscan.sourceforge.net) [31], [32] mpileup2cns tool which produces an IUPAC ambiguity code for each position of the reference sequences for which the quality constraints are satisfied. We selected conservative constraints such that a position must have an overall coverage depth of 15x to be considered at all. Reads must have an read quality of 25 or more at the position of the variant to be considered. In order to call a variant, it must be supported by 20% of the reads that cover it and in lower coverage cases a minimum of 5 reads must support it. These two settings specifically should serve to minimize the likelihood of erroneous calls from sequencing errors. Further, a p-value (calculated via Fisher’s Exact Test and indicates the likelihood of the call) must be below 0.01. Lastly a strand filter is applied to help identify variants that could be the result of PCR over-amplification. Full command line parameters and a flow chart describing this process are in Protocol S1.

### Finding Discriminatory SNPs

The consensus calls for the three parental strains were compared and any position where one strain was found to be different from the other two strains was noted. In order for a position to be considered different in one strain, that strain had to be homozygous for the difference, and the observed nucleotides in the other strains had to be supported by the same constraints as were described in the SNP calling section above. The discriminatory SNPs (dSNPs) were then annotated with Ensembl transcript and gene IDs and grouped by transcript. Full command line parameters and a flow chart describing this process are in Protocol S1.

### Determining ASE

Two main steps are required to enable the determination of ASE values. The first step involves normalizing to control for sample size variations and scaling coverage. The first step appropriately scales overall transcript expression values to enable comparisons between samples. In the second step the values of discriminating SNPs are integrated to estimate the contribution of each genome to the overall expression of a given gene.

Short reads from triploid samples were mapped using the STAR aligner, and the resulting SAM output was converted to mpileup format as previously described (Protocol S1). Then a perl script was used to extract the depth of coverage for each position in the reference sequences in each sample, and calculate the geometric mean of the depth of coverage for each transcript as a measure of the expression value. Only positions for which the coverage depth is greater than 0 are considered, therefore we define the covered length 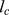 to be the subset of positions in the transcript that have a coverage depth of 1 or greater. The geometric mean of the coverage depth over each transcript (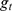) with covered length (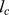) and coverage depth at each position (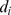) is described below.
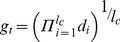
We then use the geometric mean of coverage depth in a transcript as a measure of transcript expression.

Because the amount of data generated varies by sequencing lane and sample, we next normalized the data to control for variations in overall sample size. Briefly, the geometric mean of each transcript expression value across samples is taken as a representative transcript expression value. Then the transcript expression values in each sample are divided by their associated representative expression values. The median of these quotients in a given sample is then taken as the size factor with which to adjust each individual transcript expression value in that sample. This method is the same as is described by the authors of the DESeq package [33] with the exception that instead of total read counts here we use the geometric mean of read depth as our transcript expression value.

Next the fractional expression is measured at dSNP sites. This simply involves identifying what fraction of reads covering a dSNP position are attributed to the strain identified by that dSNP then multiplying that by the geometric mean of the coverage depth.

The fractional expression (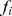) at position 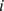 for a given strain 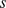 is given by the following formula:
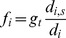
where 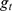 is the geometric mean of coverage depth in the transcript, 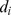 is the number of reads covering position 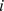, and 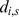 is the number of reads covering position 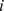 that are attributable to strain 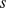.

Only transcripts that have at least one dSNP representative of each allele are considered. The fractional expression values determined for all of the dSNPs of a given allele for a given transcript are averaged to determine that allele-specific expression (ASE) value. Only values greater than zero are considered for the signal averaging, but if all expression values are zero then that is the reported value. Full command line parameters and a flow chart describing this process are in Protocol S1.

### Parental dSNP and Hybrid ASE Validation

From the list of transcripts identified as presenting informative dSNPs, seven were selected for further analysis: ENSORLT00000001009; ENSORLT00000013099; ENSORLT00000024856; ENSORLT00000008958; ENSORLT00000014111; ENSORLT00000013388; ENSORLT00000012489. The selection of these transcripts was based on the allelic expression patterns observed in the tri-hybrids. The selected targets are representative of different extreme possibilities of allelic usage in this “three allelic” context (Figure S1). Specific primers for each of these transcripts (Table S1) were designed based on the sequences alignment of the three alleles (OR, HNI-I, SOK) with Bioedit v7.2.0.

From an aliquot of each RNA sample, first-strand cDNA was synthesized with RevertAid First Strand cDNA Synthesis Kit (Fermentas). Amplification of each target transcript was performed for each sample (Table S1) according to the following PCR conditions: pre-heating at 95°C for 5 min, 35 cycles at 95°C for 30 s, 55°C or 57°C for 30 s and 72°C for 45 s and a final extension at 72°C for 10 min. The PCR products were Sanger sequenced and the sequences analyzed (Sequencher ver. 4.0, Gene Codes Corporation, Inc.) in order to validate the SNP calling between the three parental lines and the presence of expression derived from any single allele, any two, or all three alleles in the tri-hybrids.

### Synthetic Data Set

The synthetic data were generated to match the calculated ASE values of trpA. The total number of reads generated per transcript were the same as found by aligning short reads from trpA to the reference. The fraction derived from each strain was determined by multiplying this total, by the fraction of total expression attributed to each strain. For example, if for a given transcript, the ASE values of HNI-II, OR, and SOK were 10, 20, and 30 respectively and the total number of fragments aligned to that transcript were 500, then the number of reads generated for HNI-II, OR, and SOK variants would be 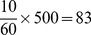, 
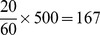
, and 
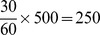
 respectively. In order to generate the fragments contributed by each strain we created strain-specific reference sequences with the strain-specific SNPs substituted in to the reference transcript sequence. Then the required number of fragments were generated as paired 100 bp reads with a fragment size of 250 bp taken at randomized start positions along the transcript length. This short read data set was then analyzed using the same software pipeline.

## Supporting Information

Figure S1 **Genes selected for dSNP confirmation.** Genes selected for confirmation of dSNPs were taken from all 4 quadrants and are shown here as red circles overlaid on a background of grey points showing the change in gene expression vs. the dispersion of allele specific expression in trpA. The horizontal axis indicates differential expression of the triploids with respect to the parent fish lines. The average of whole gene expression in trpA and trpB is compared to the average of the 3 parent species. The vertical axis of indicates the coefficient of variation (c_v_) of ASE values for each transcript in trpA. c_v_ values near 0 indicate that the three alleles are expressed at near equal levels, and increasing c_v_ values indicate a greater dispersion of allele-specific expression.
(TIF)

Figure S2 **Stacked histograms of c_v_ values in all allelic imbalance categories.** Stacked histograms of coefficient of variation of allele expression values in transcripts grouped by allele imbalance categories. A c_v_ value near 0.87 is consistent with complete suppression of one allele. This shows the clear preference for HNI-II and OR silencing (spike in bin of c_v_ values covering 0.85 to 0.90).
(TIF)

Table S1 **Primers for dSNP validation.** Primers used for validation of dSNPs by Sanger sequencing are listed along with the gene and transcript IDs and other descriptive information from ENSEMBL version 65. The quadrants listed are a reference to those defined in Figure 6.
(XLSX)

Table S2 **Confirmation of dSNPs by Sanger sequencing.** This table lists the Sanger sequencing results of dSNP sites from seven transcripts and whether or not they confirm the consensus nucleotide calls made by VarScan using RNAseq data. In total 32 out of 36 dSNPs are confirmed. The four misses were shown to be heterozygous in the parental strains by Sanger sequencing.
(XLSX)

Table S3 **Allele specific expression values.** This table lists allele specific expression values for trpA and trpB and whole gene expression for the three parental line samples as calculated by our methods. The non-bold text in blue, green, and red colored cells lists the expression values detected for HNI-II, OR, and SOK alleles respectively for trpA and trpB. The bold text lists whole gene expression values for trpA, trpB, HNI-II, OR, and SOK as calculated by our software pipeline. The whole gene expression values are the sum of allele-specific expression for each transcript. ENSEMBL transcript IDs are used to identify each transcript.
(TXT)

Protocol S1 **Flowcharts and command lines for software tools used.** An extensive set of flow charts with command line options used for our analysis. This includes several custom perl scripts which are available upon request.
(PDF)

Text S1 **Tests to determine association of several factors with low accuracy of ASE.** A short summary of extra tests done to investigate possible causes of low accuracy of ASE measurements.
(DOCX)
