# Supplementary figures and images for: Novel Method for Analysis of Allele Specific Expression in Triploid Oryzias latipes Reveals Consistent Pattern of Allele Exclusion
Source: PLoS One. 2014 Jun 19;9(6):e100250. doi: 10.1371/journal.pone.0100250 (PMC4063754; doi:10.1371/journal.pone.0100250)

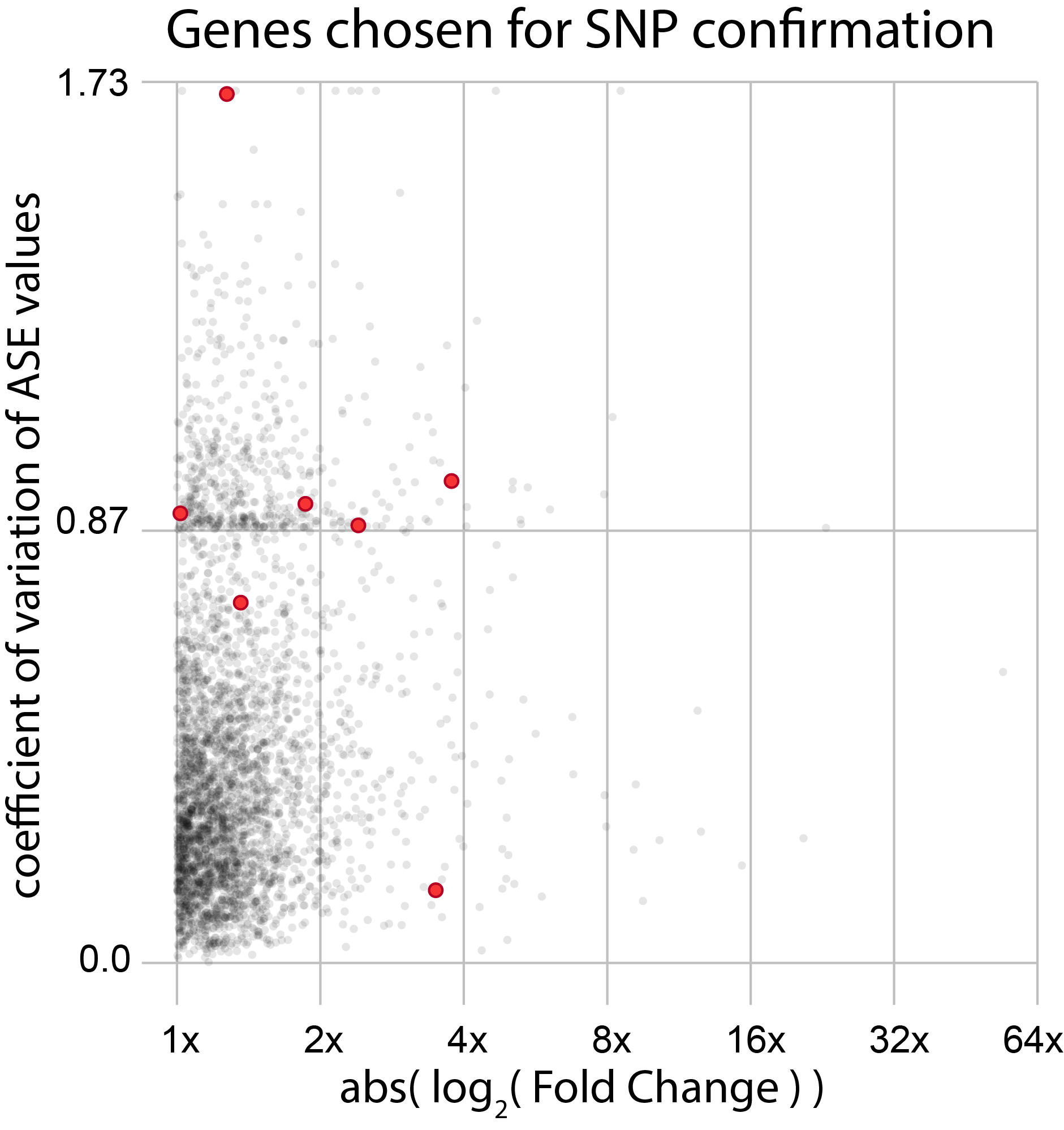

Supplement: Figure S1 — Genes selected for dSNP confirmation. Genes selected for confirmation of dSNPs were taken from all 4 quadrants and are shown here as red circles overlaid on a background of grey points showing the change in gene expression vs. the dispersion of allele specific expression in trpA. The horizontal axis indicates differential expression of the triploids with respect to the parent fish lines. The average of whole gene expression in trpA and trpB is compared to the average of the 3 parent species. The vertical axis of indicates the coefficient of variation (cv) of ASE values for each transcript in trpA. cv values near 0 indicate that the three alleles are expressed at near equal levels, and increasing cv values indicate a greater dispersion of allele-specific expression. (TIF) [file pone.0100250.s001.tif]

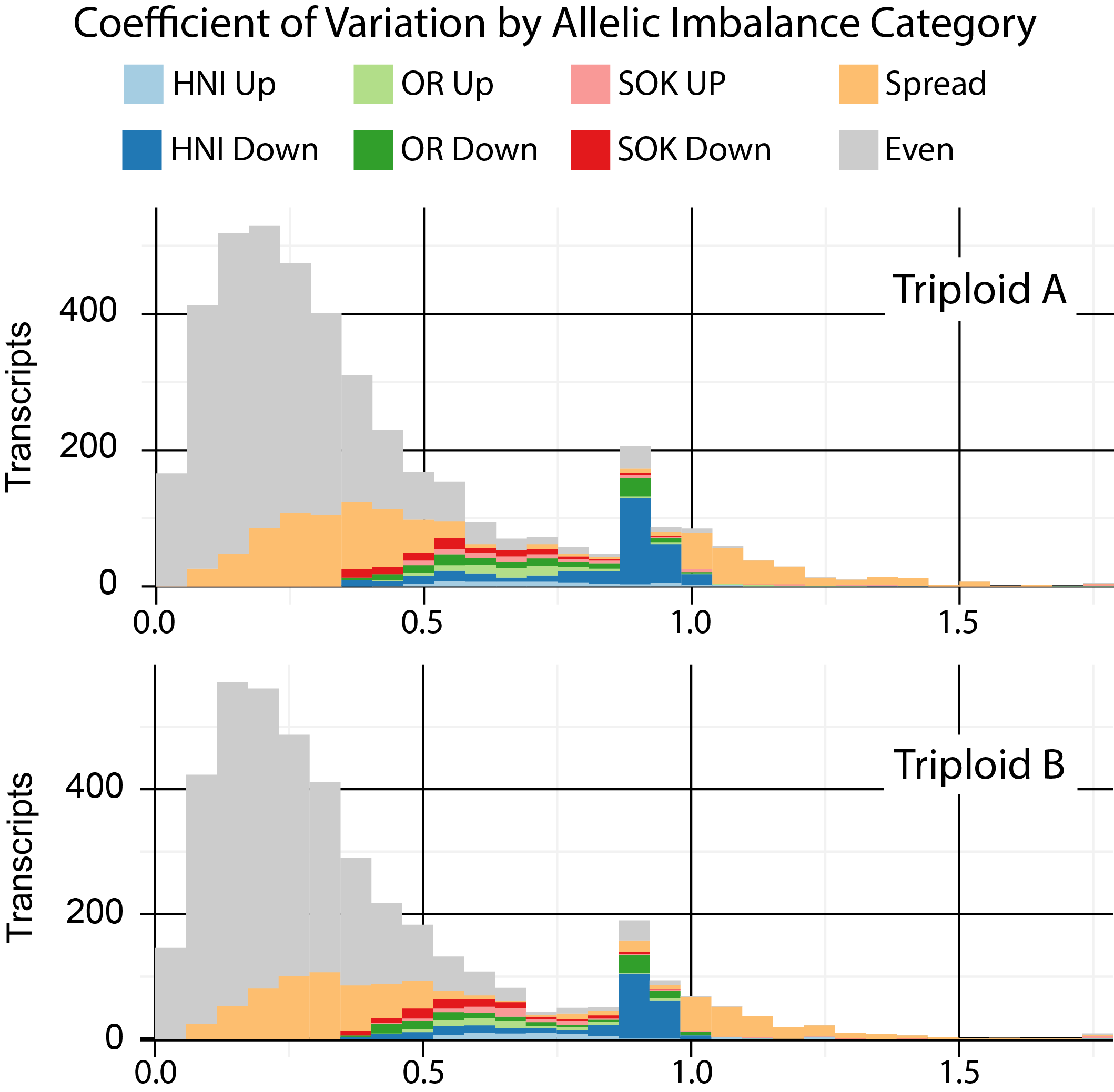

Supplement: Figure S2 — Stacked histograms of cv values in all allelic imbalance categories. Stacked histograms of coefficient of variation of allele expression values in transcripts grouped by allele imbalance categories. A cv value near 0.87 is consistent with complete suppression of one allele. This shows the clear preference for HNI-II and OR silencing (spike in bin of cv values covering 0.85 to 0.90). (TIF) [file pone.0100250.s002.tif]
