# Supplementary material for: Novel Method for Analysis of Allele Specific Expression in Triploid Oryzias latipes Reveals Consistent Pattern of Allele Exclusion
Source: PLoS One. 2014 Jun 19;9(6):e100250. doi: 10.1371/journal.pone.0100250 (PMC4063754; doi:10.1371/journal.pone.0100250)
Supplement: Protocol S1 — Flowcharts and command lines for software tools used. An extensive set of flow charts with command line options used for our analysis. This includes several custom perl scripts which are available upon request. (PDF) [file pone.0100250.s006.pdf]

# Short read alignment

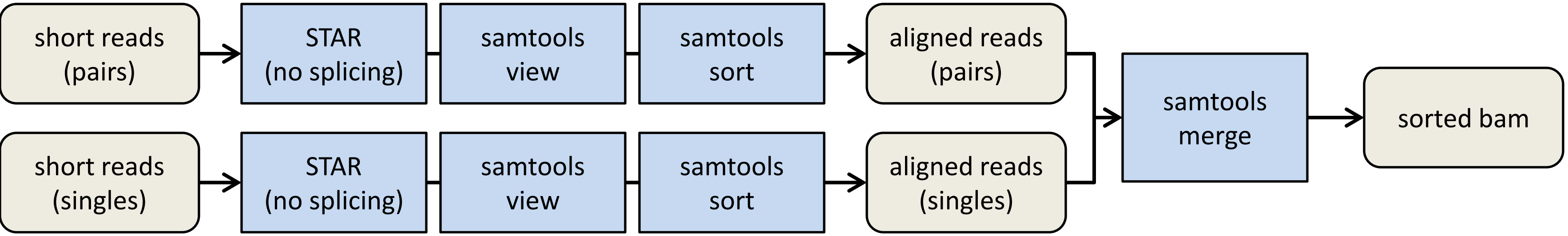


```
## Align paired reads
STAR \
--runMode alignReads \
--genomeLoad LoadAndKeep \
--genomeDir ~/stardb/O.latipes_cDNA \
--readFilesIn /path/to/reads/reads_1.fastq /path/to/reads/reads_2.fastq \
--runThreadN $THREADS \
--outFileNamePrefix paired/ \
--outFilterMismatchNoverLmax 0.05 \
--outFilterMultimapNmax 10  \
--alignIntronMax 20 \ # these two together will
--alignIntronMin 21 \ # disable splicing in STAR
--outSAMattributes All \
--outStd SAM | \
samtools view -S -h -b -u - | samtools sort -@ $THREADS - paired/paired.sorted

## Align single reads
STAR \
--runMode alignReads \
--genomeLoad LoadAndKeep \
--genomeDir ~/stardb/O.latipes_cDNA \
--readFilesIn /path/to/reads/reads_S.fastq \
--runThreadN $THREADS \
--outFileNamePrefix single/ \
--outFilterMismatchNoverLmax 0.05 \
--outFilterMultimapNmax 10  \
--alignIntronMax 20 \ # these two together will
--alignIntronMin 21 \ # disable splicing in STAR
--outSAMattributes All \
--outStd SAM | \
samtools view -S -h -b -u - | samtools sort -@ $THREADS - single/single.sorted

## Merge sorted aligned reads into one bam file
samtools merge merged.sorted.bam paired/paired.sorted.bam single/single.sorted.bam
```

# Identify SNPs

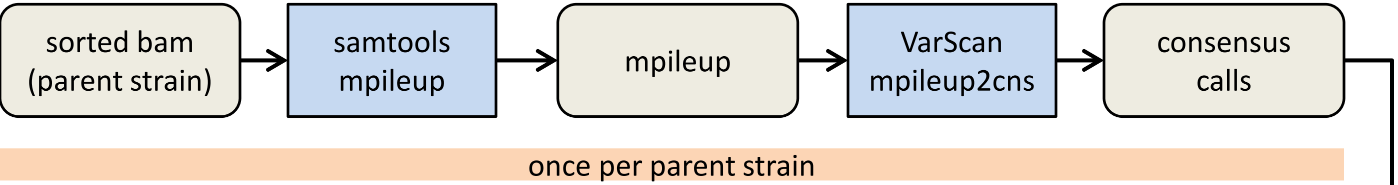


```
samtools mpileup -B -f reference.fasta sorted.bam > strainA.mpileup
java -jar VarScan.v2.3.6.jar mpileup2cns strainA.mpileup \
--min-coverage 15 \
--min-reads2 5 \
--min-avg-qual 25 \
--min-var-freq 0.20 \
--p-value 0.01 \
--strand-filter 1 \
> strainA.mpileup.cns
```

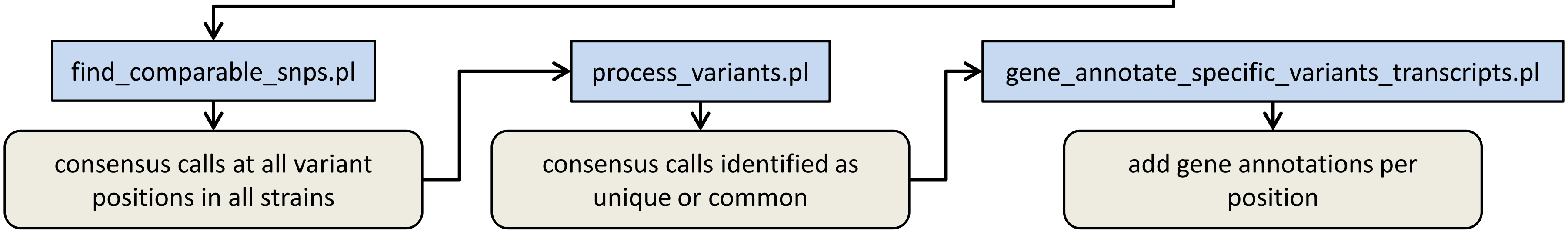


```
# Identify variants in all strains and consensus calls (as IUPAC ambiguity codes) at the same position in all parental strains
find_comparable_snps.pl reference.fasta \
strainA strainA.mpileup.cns \
strainB strainB.mpileup.cns \
strainC strainC.mpileup.cns \
> strainA_strainB_strainC.comp.cns

# Determine whether variants are uniquely found in one or more strains or whether the observed nucleotide at a given
# position and in a given strain are also seen in that position in other strains.
process_variants.pl --consensus-file=strainA_strainB_strainC.comp.cns > strainA_strainB_strainC.specific.variants

# Add annotations identifying ENSEMBL gene ID from ENSEMBL gene annotation in GTF format
gene_annotate_specific_variants_transcripts.pl genes.gtf strainA_strainB_strainC.specific.variants \
> strainA_strainB_strainC.ENSG.specific.variants
```

## Normalize for sample size

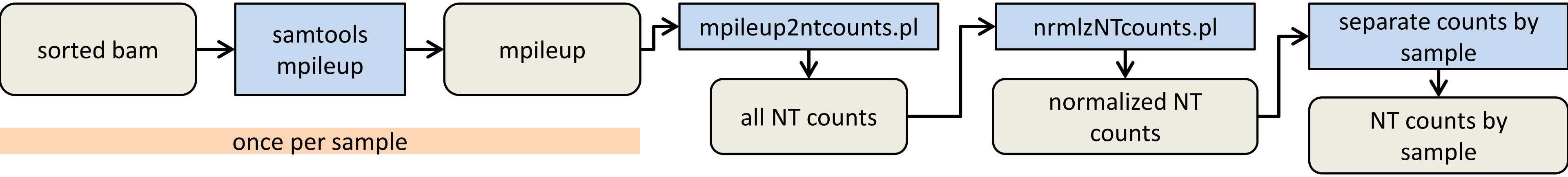


```
# Once for each sample: use samtools to generate an mpileup file from the sorted bam
samtools mpileup -B -f refseq.fasta sample.sorted.bam > sample.mpileup

# Count occurrences of each nucleotide at each position in all samples and report as large tabular matrix
mpileup2ntcounts.pl strainA.mpileup strainB.mpileup strainC.mpileup \
triploidA.mpileup triploidB.mpileup triploidC.mpileup > all.ntcounts

# Normalize counts for sample size
nrmlzNTCounts.pl all.ntcounts > all_nrml_ntcounts2

#Separate normalized counts by sample
perl -e '$a=`head -n 1 all_nrml_ntcounts2`;
chomp $a;
@b=split/\t/,$a;
$n=(@b-2)/4-1;
for $i (0..$n) {
		$s=$i*4+3;
		$e=$s+3;
		$name=$b[$s-1];
		$name=~s/A\.(\S+)/$1/;
		@args=("cut -f ".join(",",1,2,$s..$e)." all_nrml_ntcounts2 > $name.nrml.ntcounts");
		system @args;
}'
```

## Integrate normalized NT counts to calculate ASE

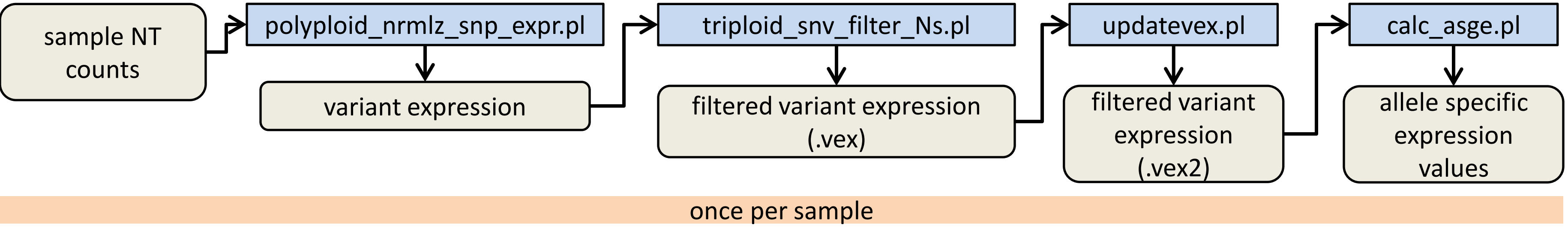


```
# Determine the expression attributable to each allele from the normalized nucleotide counts and expected nucleotides
#   at each dSNP position identified
polyploid_nrmlz_snp_expr.pl --variants= strainA_strainB_strainC.specific.variants --ntcounts=sample.nrml.ntcounts

# Filter out less informative nucleotide positions (0 counts, completely ambiguous, all NTs same, no expression expected)
triploid_snv_filter_Ns.pl --N --nonrep-zero --uncertain --all-same sample.vex > sample.f.vex

# Convert format
updatevex.pl sample.f.vex > sample.f.vex2

# Combine data from useful variant sites into per-gene allele specific expression values.
calc_asge.pl --vex2=sample.f.vex2
```
