# Supplementary material for: Novel Method for Analysis of Allele Specific Expression in Triploid Oryzias latipes Reveals Consistent Pattern of Allele Exclusion
Source: PLoS One. 2014 Jun 19;9(6):e100250. doi: 10.1371/journal.pone.0100250 (PMC4063754; doi:10.1371/journal.pone.0100250)
Supplement: Text S1 — Tests to determine association of several factors with low accuracy of ASE. A short summary of extra tests done to investigate possible causes of low accuracy of ASE measurements. (DOCX) [file pone.0100250.s007.docx]

Tests to determine association of several factors with low accuracy of ASE

A plot of total gene expression in calculated vs original data sets with points colored by correlation value shows no obvious pattern. Low and high correlation points are scattered evenly across the distribution


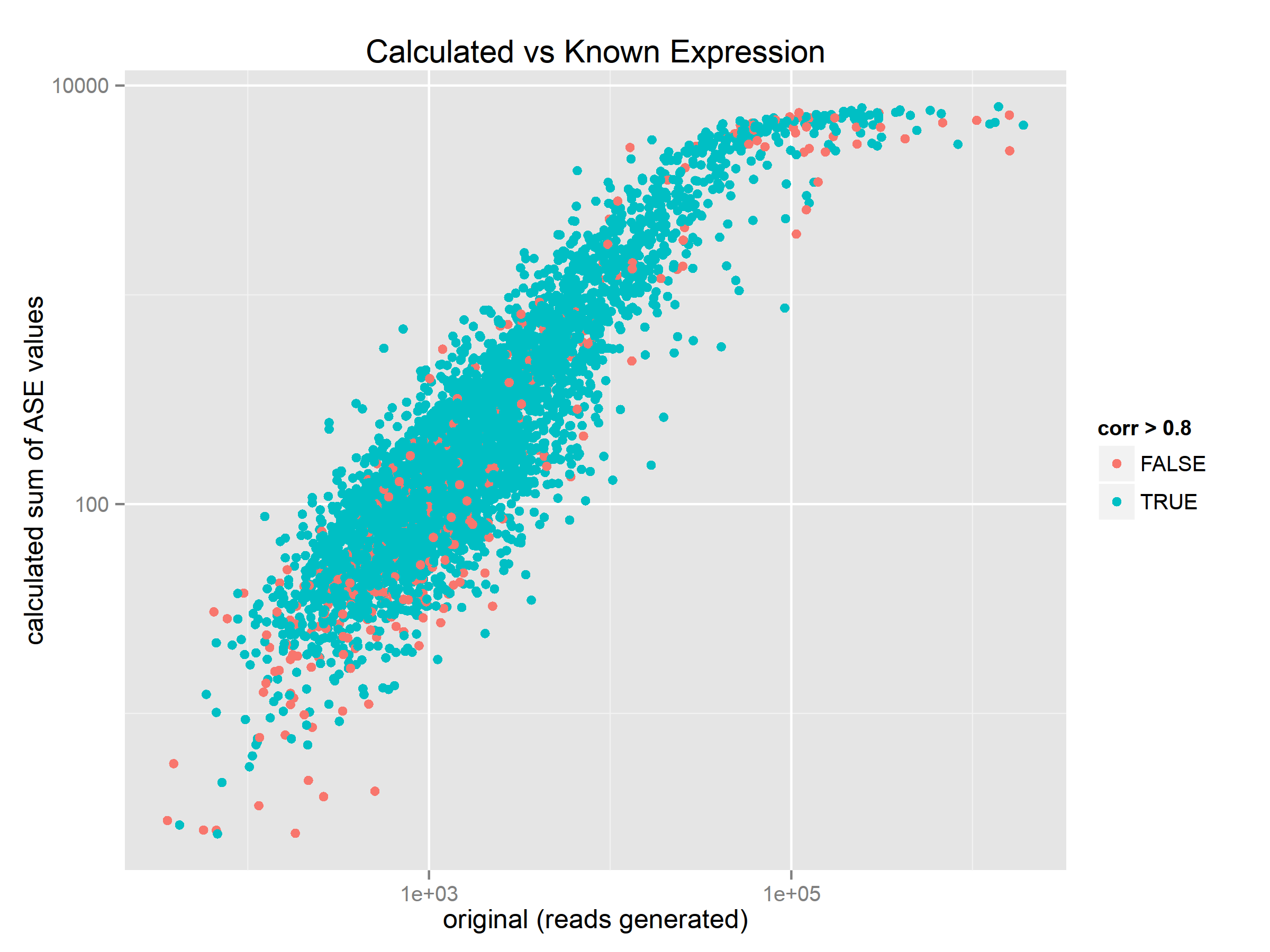


We next examined the effect of transcripts with low dSNP counts. We determined the count of dSNPs representative of the allele with fewest dSNPs for each transcript and called this value a dSNP.min. Then we split transcripts into two groups: those with a correlation coefficient of less than 0.8, and those with one of greater than or equal to 0.8. We then observed the distribution of min.snp (minimum dSNP count) in each group as a histogram. The distributions are not obviously different.


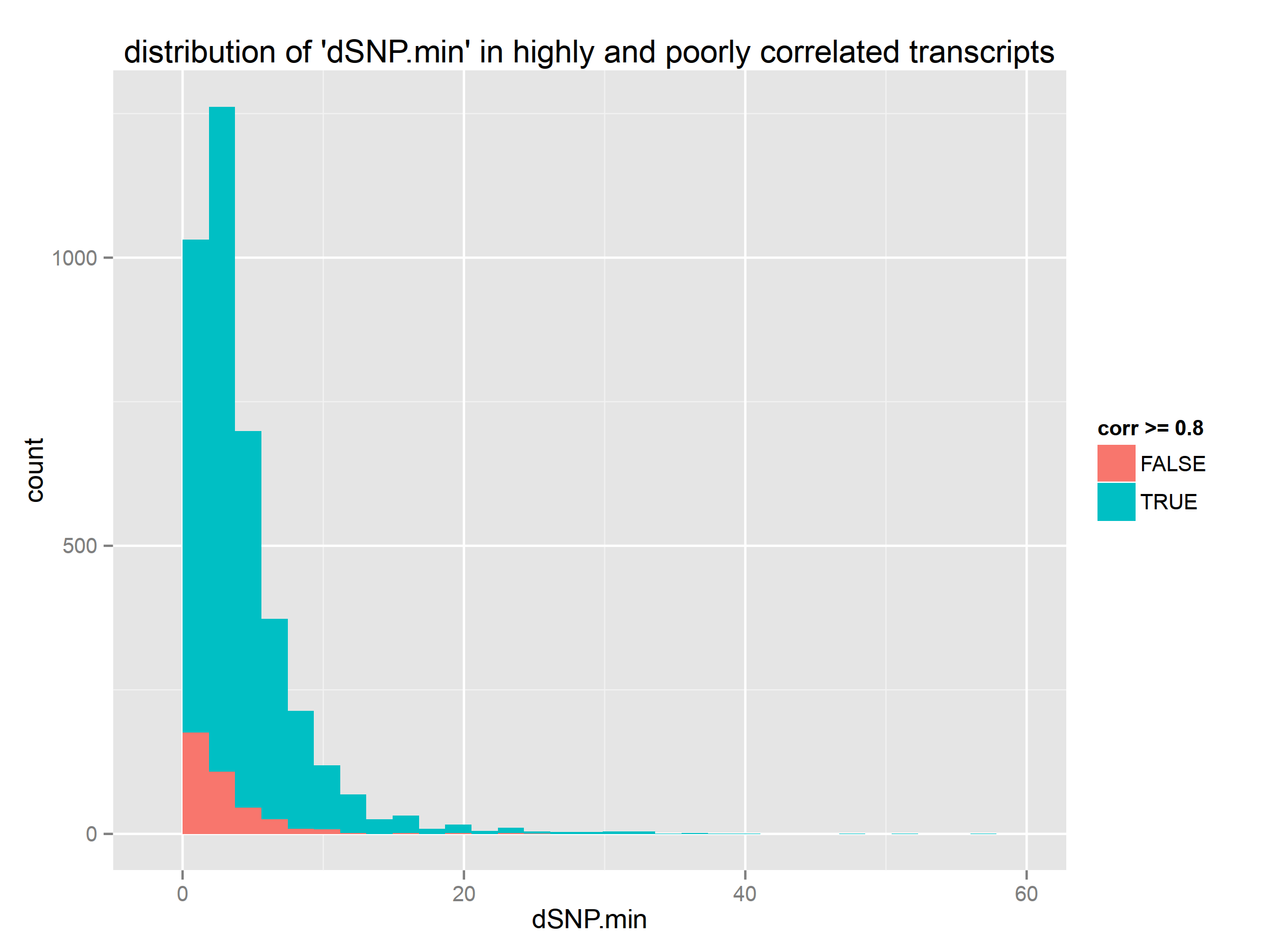


We also examined whether the *mappability* (subsequences which are highly similar to others in the reference set) played a role. We used the GEM-mappability program from the GEM toolkit (ref) to identify sub-sequences with a higher frequency within the reference set. We searched for 31bp regions and processed the output to identify the highest *mappability* score in any subsequence of a given reference transcript. The table below shows the distribution of peak *mappability* scores in the set of transcripts which had low correlation to the original data. The higher the score the more likely it would be that a read aligner could have trouble aligning to at least part of the target sequence. The majority of these problematic transcripts do not get above a *mappability* score of 3 so this does not seem to be a major factor in general.

|  | **peak *mappability* score** | | | | | | | | |  |  |
| --- | --- | --- | --- | --- | --- | --- | --- | --- | --- | --- | --- |
|  | **1** | **2** | **3** | **4** | **5** | **6** | **7** | **8** | **47** | | **83** |
| Frequency | 165 | 132 | 50 | 17 | 7 | 5 | 2 | 1 | 1 | | 1 |
